# Supplementary material for: Cohort profile: the PHARMO Perinatal Research Network (PPRN) in the Netherlands: a population-based mother–child linked cohort
Source: BMJ Open. 2020 Sep 25;10(9):e037837. doi: 10.1136/bmjopen-2020-037837 (PMC7520848; doi:10.1136/bmjopen-2020-037837)
Supplement: Supplementary data [file bmjopen-2020-037837supp001.pdf]

Appendix 1. Citation list for work published on the PPRN (excluding conference abstracts)

- Houweling LM, et al.: First year of life medication use and hospital admission rates: premature compared with term infants. *J Pediatr* 163(1): 61-6.e1, 2013.
- Zomerdijk IM, et al.: Isotretinoin exposure during pregnancy: a population-based study in The Netherlands. *BMJ Open* 4(11): e005602, 2014.
- Zomerdijk IM, Houweling L. Uitgifte van mogelijk teratogene geneesmiddelen voorafgaand aan en tijdens de zwangerschap. Perinatale Registratie Nederland. Grote Lijnen 1999 - 2012. 2014:p74-75.
- Kool-Houweling LM, et al.: Infants born before 32 weeks of gestation or with respiratory disease are most likely to receive palivizumab in the Netherlands. *Acta Paediatr* 104(9): 927-32, 2015
- Zomerdijk IM, et al.: Dispensing of potentially teratogenic drugs before conception and during pregnancy: a population-based study. *Bjog* 122(8): 1119-29, 2015.
- Houben E, et al.: Increased risk of morbidities and health-care utilisation in children born following preterm labour compared with full-term labour: A population-based study. *J Paediatr Child Health*, 2018.
- Molenaar NM, et al.: Dispensing patterns of selective serotonin reuptake inhibitors before, during and after pregnancy: a 16-year population-based cohort study from the Netherlands. *Arch Womens Ment Health*, 2019
