# Supplementary material for: Cohort profile: the PHARMO Perinatal Research Network (PPRN) in the Netherlands: a population-based mother–child linked cohort
Source: BMJ Open. 2020 Sep 25;10(9):e037837. doi: 10.1136/bmjopen-2020-037837 (PMC7520848; doi:10.1136/bmjopen-2020-037837)
Supplement: Author's manuscript [file bmjopen-2020-037837.draft_revisions.pdf]

BMJ Open is committed to open peer review. As part of this commitment we make the peer review history of every article we publish publicly available.

When an article is published we post the peer reviewers' comments and the authors' responses online. We also post the versions of the paper that were used during peer review. These are the versions that the peer review comments apply to.

The versions of the paper that follow are the versions that were submitted during the peer review process. They are not the versions of record or the final published versions. They should not be cited or distributed as the published version of this manuscript.

BMJ Open is an open access journal and the full, final, typeset and author-corrected version of record of the manuscript is available on our site with no access controls, subscription charges or pay-per-view fees (http://bmjopen.bmj.com).

If you have any questions on BMJ Open's open peer review process please email info.bmjopen@bmj.com

BMJ Open

Cohort Profile: the PHARMO Perinatal Research Network (PPRN) in the Netherlands: a population-based mother-child linked cohort

Journal:	*BMJ Open*
Manuscript ID	bmjopen-2020-037837
Article Type:	Cohort profile
Date Submitted by the Author:	26-Feb-2020

Complete List of Authors:	Houben, Eline; PHARMO Institute for Drug Outcomes Research; Erasmus MC, Department of Obstetrics and Gynaecology<br>Broeders, Lisa; Perined<br>Steegers, Eric; Erasmus MC, Department of Obstetrics and Gynaecology<br>Herings, Ron; PHARMO Institute; Amsterdam UMC, Department of Epidemiology & Biostatistics

Keywords:	CLINICAL PHARMACOLOGY, EPIDEMIOLOGY, GYNAECOLOGY, NEONATOLOGY, OBSTETRICS, PAEDIATRICS

SCHOLARONE™
Manuscripts

I, the Submitting Author has the right to grant and does grant on behalf of all authors of the Work (as defined in the below author licence), an exclusive licence and/or a non-exclusive licence for contributions from authors who are: i) UK Crown employees; ii) where BMJ has agreed a CC-BY licence shall apply, and/or iii) in accordance with the terms applicable for US Federal Government officers or employees acting as part of their official duties; on a worldwide, perpetual, irrevocable, royalty-free basis to BMJ Publishing Group Ltd ("BMJ") its licensees and where the relevant Journal is co-owned by BMJ to the co-owners of the Journal, to publish the Work in this journal and any other BMJ products and to exploit all rights, as set out in our licence.

The Submitting Author accepts and understands that any supply made under these terms is made by BMJ to the Submitting Author unless you are acting as an employee on behalf of your employer or a postgraduate student of an affiliated institution which is paying any applicable article publishing charge ("APC") for Open Access articles. Where the Submitting Author wishes to make the Work available on an Open Access basis (and intends to pay the relevant APC), the terms of reuse of such Open Access shall be governed by a Creative Commons licence – details of these licences and which Creative Commons licence will apply to this Work are set out in our licence referred to above.

Other than as permitted in any relevant BMJ Author's Self Archiving Policies, I confirm this Work has not been accepted for publication elsewhere, is not being considered for publication elsewhere and does not duplicate material already published. I confirm all authors consent to publication of this Work and authorise the granting of this licence.

For peer review only

COHORT PROFILE

Cohort Profile: the PHARMO Perinatal Research Network (PPRN) in the Netherlands: a population-based mother-child linked cohort

Author list

E. Houben*[1,2], L. Broeders[3], E.A.P. Steegers[2], R.M.C. Herings[1,4]

[1] PHARMO Institute for Drug Outcomes Research, Utrecht, The Netherlands

[2] Department of Obstetrics and Gynaecology, Erasmus MC, Rotterdam, The Netherlands

[3] Perined, Utrecht, The Netherlands

[4] Amsterdam UMC, Vrije Universiteit Amsterdam, Epidemiology and Biostatistics, Amsterdam, The Netherlands

Target journal
BMJ open (Cohort Profile)

Keywords
perinatal studies, data record linkage, mother-child linkage, birth cohort, pregnancy, child health

*Corresponding author. E-mail: eline.houben@pharmo.nl

For peer review only

For peer review only - http://bmjopen.bmj.com/site/about/guidelines.xhtml

Abstract

Purpose: Evidence from clinical trials is lacking behind for pregnant women and children, as researchers are often hesitant to include these subjects due to a variety of reasons. Therefore, the PHARMO Perinatal Research Network (PPRN) was set up as a resource for life-course perinatal and paediatric research by linking population-based data from existing registrations.

Participants: From 1999-2017, the PPRN captures approximately 542,900 pregnancies of 387,100 mothers ('Pregnancy Cohort'). Additionally, mother-child linkage is currently available for a quarter of these pregnancies ('Child Cohort'). The PPRN contains preconceptional information on maternal healthcare, as well as detailed pregnancy and neonatal data, extending into long-term follow-up and outcomes after birth for both mother and child up to nearly twenty years. It includes linked data from different primary and secondary healthcare settings.

Findings to date: Through record linkage of the Netherlands Perinatal Registry and the PHARMO Database Network, we have established a large population-based research network including data on demographics, medication use, medical conditions and details concerning labour, birth and neonatal outcomes. Here, we provide an overview of record types available from the PPRN, available database follow-up and pregnancy characteristics of the PPRN cohorts. The PPRN has been used for a number of different pharmacoepidemiological studies, for example to confirm that preterm born infants were more likely than full-term infants to be hospitalised or use medication. Similar long-term comparisons showed that children born following spontaneous preterm labour were at increased risk of neurodevelopmental and respiratory conditions. Most recently, the PPRN provided important evidence on the trends in use of potentially harmful medication during pregnancy.

Future plans: The PPRN provides a unique and rich data set facilitating large-scaled observational pharmacoepidemiological perinatal research. The patient-level linkage of many different healthcare data sources allows for long-term follow-up of mother and child, with ongoing annual updates.

For peer review only

For peer review only - http://bmjopen.bmj.com/site/about/guidelines.xhtml

Introduction

Evidence from clinical trials is often lacking behind for pregnant women and children, as researchers are often hesitant to include these subjects due to a variety of reasons.[1,2] Therefore, the PHARMO Perinatal Research Network (PPRN) was set up as a resource for life-course perinatal and paediatric research by linking population-based data from existing registrations. It was initiated around 2010 at the PHARMO Institute for Drug Outcomes Research in collaboration with Perined. At that time it was set up to study the relation between medication exposure during pregnancy and pregnancy outcomes, but the applications of the DUBC have extended considerably over the years, along with the continuous expansion of the underlying databases.

Cohort description

Setting

The PHARMO Perinatal Research Network (PPRN) is a unique linkage of the Netherlands Perinatal Registry (Perined) and the PHARMO Database Network (PHARMO). With data collection starting in 1999, the linkage of these data sources facilitates large-scaled observational pharmacoepidemiological perinatal research. It contains preconceptional information on maternal healthcare extending into long-term follow-up and outcomes after birth for both mother and child, with ongoing annual updates of the routinely collected data.

Data sources

Perined is a nationwide registry in which medical data around pregnancy and birth is included from pregnancies with a gestational age of at least 16 weeks.[3] It is a linked database combining medical registries from four professional groups that provide perinatal care: general practitioner, midwives, gynaecologists and neonatologists/paediatricians. Among the items reported are maternal demographics and medical conditions, pregnancy complications and details concerning labour, birth and neonatal outcomes. Linking the records is a complex operation - especially when it comes to records that originate from different datasets. Probabilistic linkage based on matching data is performed in the absence of unique identification of mother and/or child. There is a firm basis for deciding whether two records describe the same case or have a lot of resemblance. The threshold value for such a decision depends on the situation and is statistically substantiated.[4] The established registry reflects virtually all deliveries in the Netherlands (~99% agreement with the Municipal Administration). The frequency of data collection and processing is four times a year. The average lag time of the data is half a year.

PHARMO is a population-based network of databases combining data from different primary and secondary healthcare settings in the Netherlands. These different data sources, including data from general practices, in- and out-patient pharmacies, clinical laboratories, hospitals, the cancer registry, pathology registry and perinatal registry, are linked on a patient level through validated algorithms. To ensure the privacy of the data in the PHARMO Database Network, the collection, processing,

For peer review only

For peer review only - http://bmjopen.bmj.com/site/about/guidelines.xhtml

linkage and anonymisation of the data is performed by the foundation “Stichting Informatievoorziening voor Zorg en Onderzoek” (STIZON). STIZON is an independent, ISO/IEC 27001 certified foundation, which acts as a Trusted Third Party (TTP) between the data sources and the PHARMO Institute. Detailed information on the methodology and the validation of the used record linkage method can be found elsewhere.[5,6] PHARMO covers approximately a quarter of the Dutch population; however data collection period, catchment area and overlap between data sources differ. The PHARMO databases are linked on an annual basis, the average lag time of the data is one year.

Perined-PHARMO linkage

STIZON also acts as a TTP for the linkage between Perined and PHARMO. This specific linkage is primarily based on the birth date of the mother and child, their gender and their ZIP codes. In case multiple possible links are established, these determinants are supplemented with hospital admission records around delivery as well as obstetrician- or gynaecologist-prescribed medication. Furthermore, home codes that indicate mother and child live on the same address are used to verify established pairs and improve linkage specificity.

Data collection

From 1999 to 2017, the PPRN captures approximately 542,900 pregnancies of 387,100 mothers for which a PHARMO-Perined link could be established (i.e. ‘Pregnancy Cohort’). Additionally, an individual mother-child linkage is currently available for a quarter of these pregnancies allowing subjects to be followed over time up to nearly twenty years after birth and studying associations with pregnancy- or neonatal-specific outcomes (i.e. ‘Child Cohort’). A schematic overview of data captured in the PPRN and how these two cohorts interrelate is included in Figure 1 and Table 1. Further characterisation of the PPRN is included in Table 2, including the total Perined population as a reference. Details on the available database history and follow-up for the children included in the Child Cohort are presented in Figure 2.

Patient and public involvement

No patients were involved in the described linkage between exisiting registries providing an anonymous data set.

<Figure 1 here>

For peer review only

For peer review only - http://bmjopen.bmj.com/site/about/guidelines.xhtml

Table 1. Overview of the record types available from the PPRN

Record type	Description	Data availability
**Pregnancy/neonatal**	Maternal and neonatal characteristics and perinatal care from pregnancies with a gestational age of at least 16 weeks captured by midwife practices, gynaecologists, paediatricians and neonatologists (maintained by Perined).	From 1999 onwards (full coverage for PPRN cohorts)
**Medication**	General practitioner or specialist prescribed healthcare products including information on type of product, date, strength, dosage regimen, route of administration, prescriber specialty and costs dispensed by out-patient pharmacy. In-patient medication available for a subcohort.	From 1998 onwards (full coverage for PPRN cohorts for out-patient medication; in-patient medication only for a subcohort)
**General practitioner**	Patient records registered by general practitioners including information on diagnoses and symptoms, laboratory test results, general practitioner visits and referrals to specialists.	From 2003 onwards (partial coverage for PPRN cohorts)
**Laboratory tests**	Results of laboratory tests performed on clinical specimens, requested by general practitioners or medical specialists including information on date and time of testing, test result, unit of measurement and type of clinical specimen.	From 1998 onwards (partial coverage for PPRN cohorts)
**Hospital admissions**	Hospital admissions (i.e. in-patient hospital records) for more than 24 hours and admissions for less than 24 hours for which a bed is required including information on hospital admission and discharge dates, discharge diagnoses and procedures (maintained by the Dutch Hospital Data Foundation[7]).	From 1998 onwards (full coverage for PPRN cohorts up to 2015; partial coverage for PPRN cohorts from 2016 onwards) after permission is granted by the Dutch Hospital Data Foundation.
**Ambulatory care**	Ambulatory care (i.e. out-patient hospital records), including diagnosis, number of visits, involved specialist (maintained by the Dutch Hospital Data Foundation[7]).	From 2016 onwards (partial coverage for PPRN cohorts) after permission is granted by the Dutch Hospital Data Foundation.

For peer review only

For peer review only - http://bmjopen.bmj.com/site/about/guidelines.xhtml

Record type	Description	Data availability
**Pathology reports**	Data on excerpts of histological, cytological and autopsy examinations, obtained through linkage with the national Pathology Registry[8] (maintained by the PALGA Foundation).	From 1998 onwards (full coverage for PPRN cohorts) after permission is granted by the PALGA Foundation.
**Malignancies**	Data on all newly diagnosed cancer cases including information on cancer diagnosis, tumour staging, tumour site, morphology and initial treatment, obtained through linkage with the national Netherlands Cancer Registry[9] (maintained by the Netherlands Comprehensive Cancer Organisation).	From 1998 onwards (full coverage for PPRN cohorts) after permission is granted by the Netherlands Comperhensive Cancer Organisation.

For peer review only

For peer review only - http://bmjopen.bmj.com/site/about/guidelines.xhtml

Table 2. Pregnancy characteristics in the Pregnancy Cohort and the Child Cohort of the PPRN

	PHARMO Perinatal Research Network (PPRN) 1999-2017		Perined 1999-2017 (reference)
	**Pregnancy Cohort**	**Child Cohort**	**Total population**
**Number of pregnancies**	~542,900	~126,200	~3,200,000
**Number of mothers**	~387,100	~101,400	-
**Maternal characteristics**			
Age at delivery (mean ± SD; years)	31.0 ± 4.8	30.8 ± 4.7	31.0 ± 4.9
Nulliparous (%)	46	57	47
Dutch ethnicity (%)	79	84	79
Database history before delivery (mean ± SD; years)	6.0 ± 4.3	6.1 ± 4.3	-
Database follow-up after delivery (mean ± SD; years)*	7.9 ± 5.0	7.7 ± 4.7	-
**Infant characteristics**			
Male sex (%)	51	53	51
Gestational age at birth (mean ± SD; weeks)	39.1 ± 3.5	39.2 ± 2.7	39.3 ± 2.3
Preterm birth (%)	8	7	8
Multiple birth (%)	2	<0.5	4
Database follow-up after birth (mean ± SD; years)*	7.8 ± 4.7	7.8 ± 4.7	-

*Current censoring: 31 December 2018

For peer review only

For peer review only - http://bmjopen.bmj.com/site/about/guidelines.xhtml

<Figure 2 here>

Findings to date

Through record linkage of Perined and PHARMO, we have established a large population-based research network including data on demographics, medication use, medical conditions, pregnancy complications and details concerning labour, birth and neonatal outcomes. The PPRN has been used for a number of different pharmacoepidemiological studies (see Appendix 1 for a citation list of work published on the PPRN). Its applicability can be centered on the mother, the child or both (i.e. assessing the association between maternal characteristics and child outcomes). As an example, medication use during first-year of life and hospital admission rates have been assessed and compared between premature and term infants.[10] Preterm born infants were up to 2 times more likely than full-term infants to be hospitalised or use medication, especially related to respiratory disease. Similar long-term comparisons of morbidities and health-care utilisation have been made which showed that children born following spontaneous preterm labour, irrespective of gestational age at delivery or of an uncomplicated pregnancy, were at increased risk of neurodevelopmental and respiratory conditions compared with those from full-term labour pregnancies.[11] Most recently, data from the PPRN have been used to determine population-based trends over the last two decades in the use of potentially harmful medication among pregnant women (currently unpublished).

Strengths and limitations

The main strength of the PPRN lies in the ongoing assembly of detailed, population-based, anonymised data from existing registrations which makes it an invaluable resource for life-course perinatal and paediatric research. Evidence from clinical trials is often lacking behind for pregnant women and children, as researchers are often hesitant to include these subjects due to a variety of reasons, including the fear of harm to the foetus and threat of legal liability.[1,2] Therefore, the PPRN provides a very suitable, non-invasive method that stays within the many risks and objections of studies in this vulnerable population. The PPRN covers a considerable proportion of pregnancies from 1999 onwards that has been shown to reflect true estimates of the Dutch population captured in Perined[12], ensuring a high level of generalisability. The patient-level linkage of many different healthcare data sources provides a very rich dataset allowing long-term follow-up of mother and child, with data continuously being collected.

The PPRN brings together data from various sources. Data collection periods and catchment areas vary between these databases and therefore the size of the study population depends on the databases included. The 542,900 pregnancies linked in the data cut up to 2017 allow for assessment of drug use around pregnancy. Inclusion of other databases (e.g. general practitioner records or hospital admissions) will reduce the cohort size. Also, the lag time for the PHARMO-Perined linked data to become available is currently approximately one year. Furthermore, the number of records

For peer review only - http://bmjopen.bmj.com/site/about/guidelines.xhtml

that include a social security number is currently too limited to allow for deterministic record linkage between PHARMO and Perined. This availability is steadily increasing and will in the future improve the ability to differentiate between siblings in case of multiple birth pregnancies, which are now underrepresented in the Child Cohort. The current linkage methods particularly gain a high specificity and with including these unique patient identifiers the sensitivity is expected to increase further as well. The seeming underrepresentation of multiple births in the Pregnancy Cohort is caused by the fact that the presented reference proportion for the total Perined population includes all pregnancies (including terminated pregnancies and stillborns); however, comparisons by gestational age indeed show agreement between the two (data not presented). Furthermore, the higher proportion of nulliparous women in the Child Cohort compared to the other two cohorts is mainly influenced by families more often moving houses shortly after delivery of a second child compared to their first child, and due to the new address it is less likely that the child can be traced back in the PHARMO Database network.

Collaboration

Access to the PPRN is, by governance regulations of the data collection and contractually agreed between the PHARMO Institute and Perined, restricted to researchers of the PHARMO Institute, Perined and academic affiliates. Academic affiliates from universities, hospitals or other research institutes are encouraged to apply for access to the anonymised data for scientific study purposes. The data is handled in accordance with data protection, privacy regulations and ISO certification schemes. Each data request is checked against these policies and requires permission of the applicable compliance and privacy boards of both PHARMO and Perined. Permission to external databases is requested from the database holders (e.g. Dutch Hospital Data Foundation or PALGA Foundation) on a project basis. An overview of the variables included in the different databases, the terms and conditions and data application forms are available on www.pharmo.nl and www.perined.nl. Datasets are processed in SAS (SAS Institute Inc., Cary, NC, USA), but can be converted to other data formats. Only a 10% subsample of the requested data can be downloaded by the researcher from a secure FTP server; access to the full dataset can be granted to researchers guesting at the PHARMO office.

Further details

Not applicable

For peer review only - http://bmjopen.bmj.com/site/about/guidelines.xhtml

Figure legends

Figure 1. Schematic overview of data captured in the PHARMO-Perined linked PPRN

Figure 2. Proportion and number of children included in the PPRN Child cohort, with age in years at end of follow-up (current censoring: 31 December 2018)

Article summary – Strengths and limitations of this study

- The main strength of the PPRN lies in the ongoing assembly of detailed, population-based, anonymised data from existing registrations which makes it an invaluable resource for life-course perinatal and paediatric research.
- Evidence from clinical trials is often lacking behind for pregnant women and children, as researchers are often hesitant to include these subjects due to a variety of reasons. Hence, the PPRN provides a very suitable, non-invasive method that stays within the many risks and objections of studies in this vulnerable population.
- The PPRN covers a considerable proportion of pregnancies from 1999 onwards that has been shown to reflect true estimates of the Dutch population captured in Perined, ensuring a high level of generalisability.
- Data collection periods and catchment areas vary between the linked databases and therefore the size of the study population depends on the databases included.
- Currently, we rely on probabilistic linkage methods as the number of records that include a social security number is currently too limited to allow for deterministic record linkage between PHARMO and Perined.

Contributorship statement

All authors contributed to the development of this manuscript in accordance with international ethical guidelines concerning academic publications and have approved its submission to BMJ Open.

Competing interests

The authors report no conflict of interest.

Funding

No specific grant from any funding agency in the public, commercial, or not-for-profit sectors was received for this study.

Data sharing statement

Requests for sharing study data must be made on specific grounds, either (1) with the aim of corroborating the study results in the interest of public health or (2) in the context of an audit by a competent authority. Sufficient information needs to be provided to confirm that the request is made for one of the above-mentioned purposes, including a sound justification and, in case of a

For peer review only

For peer review only - http://bmjopen.bmj.com/site/about/guidelines.xhtml

request with a view to corroborate study results, a protocol on the research for which the data will be used or a plan for quality control checks, as applicable.

For peer review only

For peer review only - http://bmjopen.bmj.com/site/about/guidelines.xhtml

References

1. Sheffield JS, Siegel D, Mirochnick M, et al. Designing drug trials: considerations for pregnant women. *Clin Infect Dis* 2014; **59 Suppl 7**(Suppl 7): S437-S44. doi: 10.1093/cid/ciu709.
2. Blehar MC, Spong C, Grady C, Goldkind SF, Sahin L, Clayton JA. Enrolling pregnant women: issues in clinical research. *Women's health issues : official publication of the Jacobs Institute of Women's Health* 2013; **23**(1): e39-45. doi: 10.1016/j.whi.2012.10.003.
3. Grote Lijnen 1999 - 2012. *Stichting Perinatale Registratie Nederland* 2013.
4. Meray N, Reitsma JB, Ravelli AC, Bonsel GJ. Probabilistic record linkage is a valid and transparent tool to combine databases without a patient identification number. *Journal of clinical epidemiology* 2007; **60**(9): 883-91. doi: 10.1016/j.jclinepi.2006.11.021.
5. Herings R, Pedersen L. Pharmacy-based Medical Record Linkage Systems. In: Strom B, Kimmel S, Hennessy S, eds. Pharmacoepidemiology. 5 ed: John Wiley & Sons, Ltd.; 2012: 270-86.
6. van Herk-Sukel MP, van de Poll-Franse LV, Lemmens VE, et al. New opportunities for drug outcomes research in cancer patients: the linkage of the Eindhoven Cancer Registry and the PHARMO Record Linkage System. *Eur J Cancer* 2010; **46**(2): 395-404. doi: 10.1016/j.ejca.2009.09.010.
7. Dutch Hospital Data Foundation. www.dhd.nl.
8. Foundation P. The nationwide network and registry of histo- and cytopathology in the Netherlands. www.palga.nl (accessed 17 April 2018 2018).
9. Netherlands Comprehensive Cancer Organisation. Netherlands Cancer Registry. www.iknl.nl (accessed 17 April 2018 2018).
10. Houweling LM, Bezemer ID, Penning-van Beest FJ, Meijer WM, van Lingen RA, Herings RM. First year of life medication use and hospital admission rates: premature compared with term infants. *The Journal of pediatrics* 2013; **163**(1): 61-6.e1. doi: 10.1016/j.jpeds.2012.12.014.
11. Houben E, Smits E, Pimenta JM, Black LK, Bezemer ID, Penning-van Beest FJ. Increased risk of morbidities and health-care utilisation in children born following preterm labour compared with full-term labour: A population-based study. *Journal of paediatrics and child health* 2018. doi: 10.1111/jpc.14225.
12. Perined. Perinatale Zorg in Nederland 2016. Utrecht: Perined; 2018.

For peer review only

For peer review only - http://bmjopen.bmj.com/site/about/guidelines.xhtml

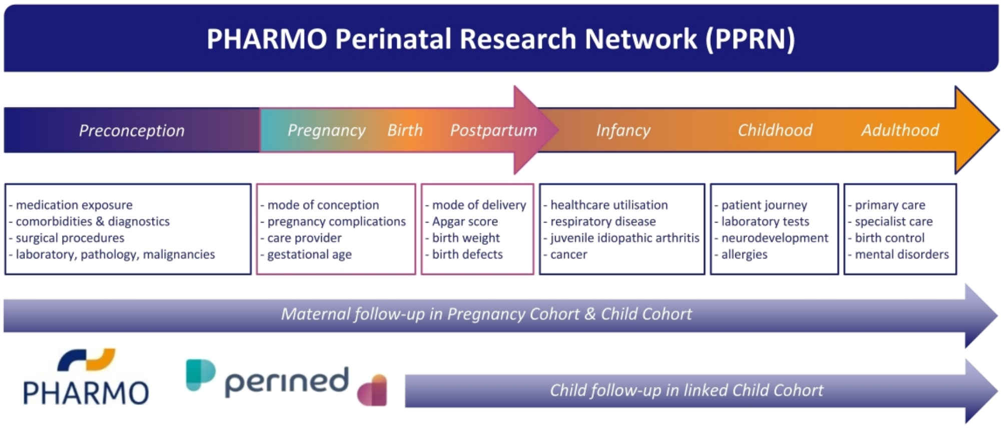

Figure 1. Schematic overview of data captured in the PHARMO-Perined linked PPRN

450x191mm (300 x 300 DPI)

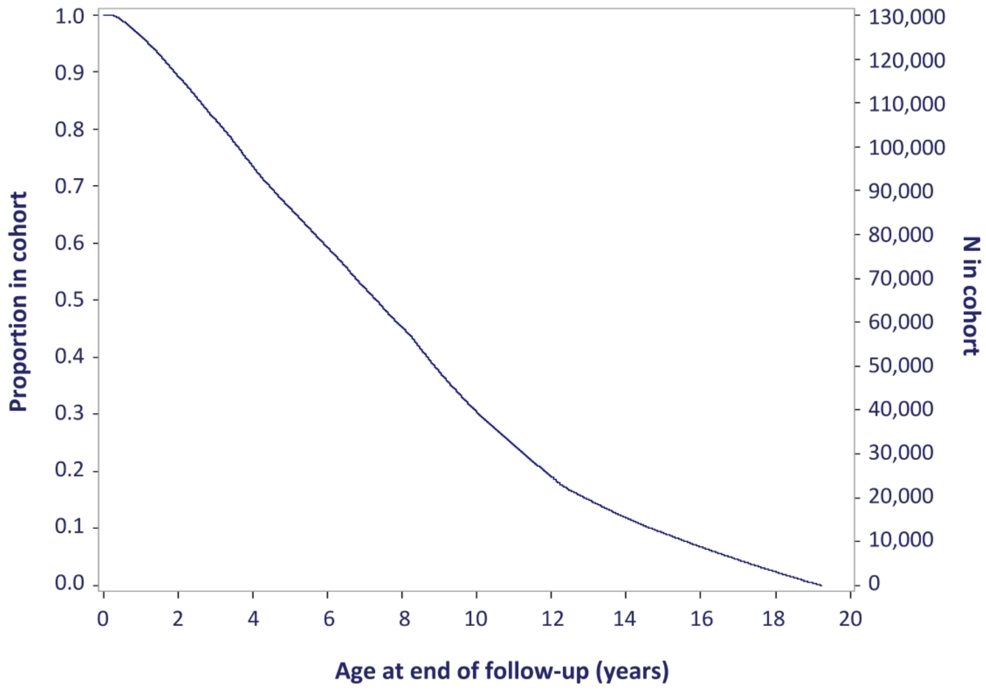

Figure 2. Proportion and number of children included in the PPRN Child cohort, with age in years at end of follow-up (current censoring: 31 December 2018)

556x396mm (300 x 300 DPI)

Appendix 1. Citation list for work published on the PPRN (excluding conference abstracts)

- Houweling LM, et al.: First year of life medication use and hospital admission rates: premature compared with term infants. *J Pediatr* 163(1): 61-6.e1, 2013.
- Zomerdijk IM, et al.: Isotretinoin exposure during pregnancy: a population-based study in The Netherlands. *BMJ Open* 4(11): e005602, 2014.
- Zomerdijk IM, Houweling L. Uitgifte van mogelijk teratogene geneesmiddelen voorafgaand aan en tijdens de zwangerschap. Perinatale Registratie Nederland. Grote Lijnen 1999 - 2012. 2014:p74-75.
- Kool-Houweling LM, et al.: Infants born before 32 weeks of gestation or with respiratory disease are most likely to receive palivizumab in the Netherlands. *Acta Paediatr* 104(9): 927-32, 2015
- Zomerdijk IM, et al.: Dispensing of potentially teratogenic drugs before conception and during pregnancy: a population-based study. *Bjog* 122(8): 1119-29, 2015.
- Houben E, et al.: Increased risk of morbidities and health-care utilisation in children born following preterm labour compared with full-term labour: A population-based study. *J Paediatr Child Health*, 2018.
- Molenaar NM, et al.: Dispensing patterns of selective serotonin reuptake inhibitors before, during and after pregnancy: a 16-year population-based cohort study from the Netherlands. *Arch Womens Ment Health*, 2019

For peer review only

BMJ Open

Cohort Profile: the PHARMO Perinatal Research Network (PPRN) in the Netherlands: a population-based mother-child linked cohort

Journal:	*BMJ Open*
Manuscript ID	bmjopen-2020-037837.R1
Article Type:	Cohort profile
Date Submitted by the Author:	08-Jul-2020
Complete List of Authors:	Houben, Eline; PHARMO Institute for Drug Outcomes Research; Erasmus MC, Department of Obstetrics and Gynaecology<br>Broeders, Lisa; Perined<br>Steegers, Eric; Erasmus MC, Department of Obstetrics and Gynaecology<br>Herings, Ron; PHARMO Institute; Amsterdam UMC, Department of Epidemiology & Biostatistics
**Primary Subject Heading**:	Epidemiology
Secondary Subject Heading:	Obstetrics and gynaecology, Paediatrics, Qualitative research, Research methods
Keywords:	CLINICAL PHARMACOLOGY, EPIDEMIOLOGY, GYNAECOLOGY, NEONATOLOGY, OBSTETRICS, PAEDIATRICS

SCHOLARONE™
Manuscripts

I, the Submitting Author has the right to grant and does grant on behalf of all authors of the Work (as defined in the below author licence), an exclusive licence and/or a non-exclusive licence for contributions from authors who are: i) UK Crown employees; ii) where BMJ has agreed a CC-BY licence shall apply, and/or iii) in accordance with the terms applicable for US Federal Government officers or employees acting as part of their official duties; on a worldwide, perpetual, irrevocable, royalty-free basis to BMJ Publishing Group Ltd ("BMJ") its licensees and where the relevant Journal is co-owned by BMJ to the co-owners of the Journal, to publish the Work in this journal and any other BMJ products and to exploit all rights, as set out in our licence.

The Submitting Author accepts and understands that any supply made under these terms is made by BMJ to the Submitting Author unless you are acting as an employee on behalf of your employer or a postgraduate student of an affiliated institution which is paying any applicable article publishing charge ("APC") for Open Access articles. Where the Submitting Author wishes to make the Work available on an Open Access basis (and intends to pay the relevant APC), the terms of reuse of such Open Access shall be governed by a Creative Commons licence – details of these licences and which Creative Commons licence will apply to this Work are set out in our licence referred to above.

Other than as permitted in any relevant BMJ Author's Self Archiving Policies, I confirm this Work has not been accepted for publication elsewhere, is not being considered for publication elsewhere and does not duplicate material already published. I confirm all authors consent to publication of this Work and authorise the granting of this licence.

For peer review only

COHORT PROFILE

Cohort Profile: the PHARMO Perinatal Research Network (PPRN) in the Netherlands: a population-based mother-child linked cohort

Author list

E. Houben*[1,2], L. Broeders[3], E.A.P. Steegers[2], R.M.C. Herings[1,4]

[1] PHARMO Institute for Drug Outcomes Research, Utrecht, The Netherlands

[2] Department of Obstetrics and Gynaecology, Erasmus MC, Rotterdam, The Netherlands

[3] Perined, Utrecht, The Netherlands

[4] Amsterdam UMC, Vrije Universiteit Amsterdam, Epidemiology and Biostatistics, Amsterdam, The Netherlands

Target journal

BMJ open (Cohort Profile)

Keywords

perinatal studies, data record linkage, mother-child linkage, birth cohort, pregnancy, child health

*Corresponding author. E-mail: eline.houben@pharmo.nl

For peer review only

For peer review only - http://bmjopen.bmj.com/site/about/guidelines.xhtml

Abstract

Purpose: Observational population-based research is a very suitable non-invasive method for studies in the vulnerable populations of pregnant women and children. . Therefore, the PHARMO Perinatal Research Network (PPRN) was set up as a resource for life-course perinatal and paediatric research by linking population-based data from existing registrations.

Participants: From 1999-2017, the PPRN captures approximately 542,900 pregnancies of 387,100 mothers ('Pregnancy Cohort'). Additionally, mother-child linkage is currently available for a quarter of these pregnancies ('Child Cohort'). The PPRN contains preconceptional information on maternal healthcare, as well as detailed pregnancy and neonatal data, extending into long-term follow-up and outcomes after birth for both mother and child up to nearly twenty years. It includes linked data from different primary and secondary healthcare settings.

Findings to date: Through record linkage of the Netherlands Perinatal Registry and the PHARMO Database Network, we have established a large population-based research network including data on demographics, medication use, medical conditions and details concerning labour, birth and neonatal outcomes. Here, we provide an overview of record types available from the PPRN, available database follow-up and pregnancy characteristics of the PPRN cohorts. The PPRN has been used for a number of different pharmacoepidemiological studies, for example to confirm that preterm born infants were more likely than full-term infants to be hospitalised or use medication. Similar long-term comparisons showed that children born following spontaneous preterm labour were at increased risk of neurodevelopmental and respiratory conditions. Most recently, the PPRN provided important evidence on the trends in use of potentially harmful medication during pregnancy.

Future plans: The PPRN provides a unique and rich data set facilitating large-scaled observational pharmacoepidemiological perinatal research. The patient-level linkage of many different healthcare data sources allows for long-term follow-up of mother and child, with ongoing annual updates.

For peer review only - http://bmjopen.bmj.com/site/about/guidelines.xhtml

Strengths and limitations of this study

- The main strength of the PPRN lies in the ongoing assembly of detailed, population-based, anonymised data from existing registrations which makes it an invaluable resource for life-course perinatal and paediatric research.
- Evidence from clinical trials is often lacking behind for pregnant women and children, as researchers are often hesitant to include these subjects due to a variety of reasons. Hence, the PPRN provides a very suitable, non-invasive method that stays within the many risks and objections of studies in the vulnerable populations of pregnant women and children.
- The PPRN covers a considerable proportion of pregnancies from 1999 onwards that has been shown to reflect true estimates of the Dutch population captured in Perined, ensuring a high level of generalisability.
- Data collection periods and catchment areas vary between the linked databases and therefore the size of the study population depends on the databases included.
- Currently, we rely on probabilistic linkage methods as the number of records that include a social security number is currently too limited to allow for deterministic record linkage between PHARMO and Perined.

For peer review only

For peer review only - http://bmjopen.bmj.com/site/about/guidelines.xhtml

Introduction

Observational population-based research is a very suitable non-invasive method for studies in the vulnerable populations of pregnant women and children. Therefore, the PHARMO Perinatal Research Network (PPRN) was set up as a resource for life-course perinatal and paediatric research by linking population-based data from existing registrations. It was initiated around 2010 at the PHARMO Institute for Drug Outcomes Research in collaboration with Perined. At that time it was set up to study the relation between medication exposure during pregnancy and pregnancy outcomes, but the applications of the PPRN have extended considerably over the years, along with the continuous expansion of the underlying databases.

Cohort description

Setting

The PHARMO Perinatal Research Network (PPRN) is a unique linkage of the Netherlands Perinatal Registry (Perined) and the PHARMO Database Network (PHARMO). With data collection starting in 1999, the linkage of these population-based data sources facilitates large-scaled observational pharmacoepidemiological perinatal research. It contains preconceptional information on maternal healthcare extending into long-term follow-up and outcomes after birth for both mother and child, with ongoing annual updates of the routinely collected data.

Data sources

Perined is a nationwide registry in which medical data around pregnancy and birth is included from pregnancies with a gestational age of at least 16 weeks (including terminated pregnancies and stillborns).[1] It is a linked database combining medical registries from four professional groups that provide perinatal care: general practitioner, midwives, gynaecologists and neonatologists/paediatricians. Among the items reported are maternal demographics and medical conditions, pregnancy complications and details concerning labour, birth and neonatal outcomes. Linking the records is a complex operation - especially when it comes to records that originate from different datasets. Probabilistic linkage based on matching data is performed in the absence of unique identification of mother and/or child. There is a firm basis for deciding whether two records describe the same case or have a lot of resemblance. The threshold value for such a decision depends on the situation and is statistically substantiated.[2] The established registry reflects virtually all deliveries in the Netherlands (~99% agreement with the Municipal Administration), i.e. including home as well as hospital births. The frequency of data collection and processing is four times a year. The average lag time of the data is half a year.

PHARMO is a population-based network of databases combining subnational data from different primary and secondary healthcare settings in the Netherlands. These different data sources, including data from general practices, in- and out-patient pharmacies, clinical laboratories, hospitals, the cancer registry, pathology registry and perinatal registry, are linked on a patient level through

For peer review only - http://bmjopen.bmj.com/site/about/guidelines.xhtml

98 validated algorithms.[3] Data are retrieved directly from the source, i.e. the electronic medical records of the healthcare providers who agree to contribute to the PHARMO Database Network. All patients registered at the contributing healthcare providers are included, unless the patient requested to opt out. To ensure the privacy of the data in the PHARMO Database Network, the collection, processing, linkage and anonymisation of the data is performed by the foundation "Stichting Informatievoorziening voor Zorg en Onderzoek" (STIZON). STIZON is an independent, ISO/IEC 27001 certified foundation, which acts as a Trusted Third Party (TTP) between the data sources and the PHARMO Institute. Detailed information on the methodology and the validation of the used record linkage method can be found elsewhere.[4,5] PHARMO covers approximately a quarter of the Dutch population and is shown to be representative of the Dutch population with regard to age and sex; however data collection period, catchment area and overlap between data sources differ. The PHARMO databases are linked on an annual basis, the average lag time of the data is one year.

Perined-PHARMO linkage

STIZON also acts as a TTP for the linkage between Perined and PHARMO. This specific linkage is primarily based on the birth date of the mother and child, their gender and their ZIP codes. In case multiple possible links are established, these determinants are supplemented with hospital admission records around delivery as well as obstetrician- or gynaecologist-prescribed medication. Furthermore, home codes that indicate mother and child live on the same address are used to verify established pairs and improve linkage specificity.

Data collection

From 1999 to 2017, the PPRN captures approximately 542,900 pregnancies of 387,100 mothers for which a PHARMO-Perined link could be established (i.e. 'Pregnancy Cohort'). Additionally, an individual mother-child linkage is currently available for a quarter of these pregnancies allowing subjects to be followed over time up to nearly twenty years after birth and studying associations with pregnancy- or neonatal-specific outcomes (i.e. 'Child Cohort'). A schematic overview of data captured in the PPRN for mothers and children and how these two cohorts interrelate is included in Figure 1 and Table 1. Further characterisation of the PPRN is included in Table 2, including the total Perined population as a reference, considering that only a subsample of the Netherlands is represented by the PHARMO Database Network. Figure 2 presents the number of pregnancies included in the Pregnancy Cohort and Child Cohort by calendar year. Details on the available database follow-up for the children included in the Child Cohort are presented in Figure 3, with end of follow-up defined by either end of database registration (i.e. patient moves out of the PHARMO catchment area), death or end of study period (December 31st, 2018), whichever occurred first.

Patient and public involvement

No patients were involved in the described linkage between existing registries providing an anonymous data set.

For peer review only - http://bmjopen.bmj.com/site/about/guidelines.xhtml

136
137 *<Figure 1 here>*
138

For peer review only

For peer review only - http://bmjopen.bmj.com/site/about/guidelines.xhtml

Table 1. Overview of the record types available from the PPRN

Record type	Description	Data availability
**Pregnancy/neonatal**	Maternal and neonatal characteristics and perinatal care from pregnancies with a gestational age of at least 16 weeks captured by midwife practices, gynaecologists, paediatricians and neonatologists (maintained by Perined).	From 1999 onwards (full coverage for PPRN cohorts)
**Medication**	General practitioner or specialist prescribed healthcare products including information on type of product, date, strength, dosage regimen, route of administration, prescriber specialty and costs dispensed by out-patient pharmacy. In-patient medication available for a subcohort.	From 1998 onwards (full coverage for PPRN cohorts for out-patient medication; in-patient medication only for a subcohort)
**General practitioner**	Patient records registered by general practitioners (gatekeeper of the Dutch healthcare system) including information on diagnoses and symptoms, laboratory test results, general practitioner visits and referrals to specialists.	From 2003 onwards (partial coverage for PPRN cohorts)
**Laboratory tests**	Results of laboratory tests performed on clinical specimens, requested by general practitioners or medical specialists including information on date and time of testing, test result, unit of measurement and type of clinical specimen.	From 1998 onwards (partial coverage for PPRN cohorts)
**Hospital admissions**	Hospital admissions (i.e. in-patient hospital records) for more than 24 hours and admissions for less than 24 hours for which a bed is required including information on hospital admission and discharge dates, discharge diagnoses and procedures (maintained by the Dutch Hospital Data Foundation[6]).	From 1998 onwards (full coverage for PPRN cohorts up to 2015; partial coverage for PPRN cohorts from 2016 onwards) after permission is granted by the Dutch Hospital Data Foundation.
**Ambulatory care**	Ambulatory care (i.e. out-patient hospital records, e.g. by pediatrician), including diagnosis, number of visits, involved specialist	From 2016 onwards (partial coverage for PPRN cohorts) after permission is granted by the Dutch Hospital Data Foundation.

For peer review only - http://bmjopen.bmj.com/site/about/guidelines.xhtml

Record type	Description	Data availability
	(maintained by the Dutch Hospital Data Foundation[6]).	
**Pathology reports**	Data on excerpts of histological, cytological and autopsy examinations, obtained through linkage with the national Pathology Registry[7] (maintained by the PALGA Foundation).	From 1998 onwards (full coverage for PPRN cohorts) after permission is granted by the PALGA Foundation.
**Malignancies**	Data on all newly diagnosed cancer cases including information on cancer diagnosis, tumour staging, tumour site, morphology and initial treatment, obtained through linkage with the national Netherlands Cancer Registry[8] (maintained by the Netherlands Comprehensive Cancer Organisation).	From 1998 onwards (full coverage for PPRN cohorts) after permission is granted by the Netherlands Comprehensive Cancer Organisation.

For peer review only

For peer review only - http://bmjopen.bmj.com/site/about/guidelines.xhtml

Table 2. Pregnancy characteristics in the Pregnancy Cohort and the Child Cohort of the PPRN

	PHARMO Perinatal Research Network (PPRN) 1999-2017		Perined 1999-2017 (reference)
	**Pregnancy Cohort**	**Child Cohort**	**Total population**
**Number of pregnancies**	~542,900	~126,200	~3,200,000
**Number of mothers**	~387,100	~101,400	-
**Maternal characteristics**			
Age at delivery (mean ± SD; years)	31.0 ± 4.8	30.8 ± 4.7	31.0 ± 4.9
Nulliparous (%)	46	57	47
Dutch ethnicity (%)	79	84	79
Database history before delivery (mean ± SD; years)	6.0 ± 4.3	6.1 ± 4.3	-
Database follow-up after delivery (mean ± SD; years)*	7.9 ± 5.0	7.7 ± 4.7	-
**Infant characteristics**			
Male sex (%)	51	53	51
Gestational age at birth (mean ± SD; weeks)	39.1 ± 3.5	39.2 ± 2.7	39.3 ± 2.3
Preterm birth (%)	8	7	8
Multiple birth (%)	2	<0.5	4
Database follow-up after birth (mean ± SD; years)*	7.8 ± 4.7	7.8 ± 4.7	-

*Current censoring: 31 December 2018

For peer review only

For peer review only - http://bmjopen.bmj.com/site/about/guidelines.xhtml

<Figure 2 here>

<Figure 3 here>

Findings to date

Through record linkage of Perined and PHARMO, we have established a large population-based research network including data on demographics, medication use, medical conditions, pregnancy complications and details concerning labour, birth and neonatal outcomes. The PPRN has been used for a number of different pharmacoepidemiological studies (see Appendix 1 for a citation list of work published on the PPRN). Its applicability can be centered on the mother, the child or both (i.e. assessing the association between maternal characteristics and child outcomes). As an example, medication use during first-year of life and hospital admission rates have been assessed and compared between premature and term infants.[9] Preterm born infants were up to 2 times more likely than full-term infants to be hospitalised or use medication, especially related to respiratory disease. Similar long-term comparisons of morbidities and health-care utilisation have been made which showed that children born following spontaneous preterm labour (irrespective of gestational age at delivery) were at increased risk of neurodevelopmental and respiratory conditions compared with those from full-term labour pregnancies.[10] Most recently, data from the PPRN have been used to determine population-based trends over the last two decades in the use of potentially harmful medication among pregnant women.[11]

Strengths and limitations

The main strength of the PPRN lies in the ongoing assembly of detailed, population-based, anonymised data from existing registrations which makes it an invaluable resource for life-course perinatal and paediatric research. Evidence from clinical trials is often lacking behind for pregnant women and children, as researchers are often hesitant to include these subjects due to a variety of reasons, including the fear of harm to the foetus and threat of legal liability.[12,13] Therefore, the PPRN provides a very suitable, non-invasive method that stays within the many risks and objections of studies in the vulnerable populations of pregnant women and children. The PPRN covers a considerable proportion of pregnancies from 1999 onwards that has been shown to reflect true estimates of the Dutch population captured in Perined[14], ensuring a high level of generalisability. The patient-level linkage of many different healthcare data sources provides a very rich dataset allowing long-term follow-up of mother and child, with data continuously being collected.

The PPRN brings together data from various sources. Data collection periods and catchment areas vary between these databases and therefore the size of the study population depends on the databases included. The 542,900 pregnancies linked in the data cut up to 2017 allow for assessment of drug use during the 9-month preconception, pregnancy and 9-month postpartum periods. Inclusion of other databases (e.g. general practitioner records or hospital admissions) will reduce the

For peer review only - http://bmjopen.bmj.com/site/about/guidelines.xhtml

cohort size. As with any database, identification of medical events is limited to data that are captured as part of the medical records or other linked data sources in daily clinical practice. These data are not primarily collected for research purposes and rely on appropriate diagnostic coding. Also, the lag time for the PHARMO-Perined linked data to become available is currently approximately one year. Furthermore, the number of records that include a social security number is currently too limited to allow for deterministic record linkage between PHARMO and Perined. This availability is steadily increasing and will in the future improve the ability to differentiate between siblings in case of multiple birth pregnancies, which are now underrepresented in the Child Cohort. The current linkage methods particularly gain a high specificity and with including these unique patient identifiers the sensitivity is expected to increase further as well. The seeming underrepresentation of multiple births in the Pregnancy Cohort is caused by the fact that the presented reference proportion for the total Perined population includes all pregnancies (including terminated pregnancies and stillborns); however, comparisons by gestational age indeed show agreement between the two (data not presented). Furthermore, the higher proportion of nulliparous women in the Child Cohort compared to the other two cohorts is mainly influenced by families more often moving houses shortly after delivery of a second child compared to their first child, and due to the new address it is less likely that the child can be traced back in the PHARMO Database network.

Collaboration

Access to the PPRN is, by governance regulations of the data collection and contractually agreed between the PHARMO Institute and Perined, restricted to researchers of the PHARMO Institute, Perined and academic affiliates. Academic affiliates from universities, hospitals or other research institutes are encouraged to apply for access to the anonymised data for scientific study purposes. The data is handled in accordance with data protection, privacy regulations and ISO certification schemes. Each data request is checked against these policies and requires permission of the applicable compliance and privacy boards of both PHARMO and Perined. Permission to external databases is requested from the database holders (e.g. Dutch Hospital Data Foundation or PALGA Foundation) on a project basis. As it concerns database research with anonymous data, no Institutional Review Board or ethics committee approval is required. An overview of the variables included in the different databases, the terms and conditions and data application forms are available on www.pharmo.nl/what-we-have/data-request-PHARMO and (in Dutch) www.perined.nl/registratie/faciliteren-onderzoek. Datasets are processed in SAS (SAS Institute Inc., Cary, NC, USA), but can be converted to other data formats. Only a 10% subsample of the requested data can be downloaded by the researcher from a secure FTP server; access to the full dataset can be granted to researchers guesting at the PHARMO office.

Further details

Not applicable

For peer review only - http://bmjopen.bmj.com/site/about/guidelines.xhtml

Figure legends

Figure 1. Schematic overview of data captured in the PHARMO-Perined linked PPRN

Figure 2. Number of pregnancies included in the Pregnancy Cohort and Child Cohort by calendar year

Figure 3. Proportion and number of children included in the PPRN Child cohort, with age in years at end of follow-up (current censoring: 31 December 2018)

Contributorship statement

EH and RH had full access to all of the data in the study and take responsibility for the integrity of the data and the accuracy of the data analysis. EH, LB, ES and RH contributed to the plan and design of the study. EH performed the data analyses and drafted the manuscript and was in charge of the study planning. EH, LB, ES and RH contributed to the interpretation of the results and critical revision of the manuscript for important intellectual content and approved the final version of the manuscript. EH and RH are the guarantors of this paper. The corresponding author attests that all listed authors meet all ICMJE authorship criteria and that no others meeting the criteria have been omitted.

Competing interests

The authors report no conflict of interest.

Funding

No specific grant from any funding agency in the public, commercial, or not-for-profit sectors was received for this study. The first study in which the linkage algorithms between PHARMO and Perined were developed was funded by Abbott.

Data sharing statement

Requests for sharing study data must be made on specific grounds, either (1) with the aim of corroborating the study results in the interest of public health or (2) in the context of an audit by a competent authority. Sufficient information needs to be provided to confirm that the request is made for one of the above-mentioned purposes, including a sound justification and, in case of a request with a view to corroborate study results, a protocol on the research for which the data will be used or a plan for quality control checks, as applicable.

For peer review only

For peer review only - http://bmjopen.bmj.com/site/about/guidelines.xhtml

References

1. Grote Lijnen 1999 - 2012. *Stichting Perinatale Registratie Nederland* 2013.
2. Meray N, Reitsma JB, Ravelli AC, Bonsel GJ. Probabilistic record linkage is a valid and transparent tool to combine databases without a patient identification number. *Journal of clinical epidemiology* 2007; **60**(9): 883-91.
3. Kuiper JG, Bakker M, Penning-van Beest FJA, Herings RMC. Existing Data Sources for Clinical Epidemiology: The PHARMO Database Network. *Clin Epidemiol* 2020; **12**: 415-22.
4. Herings R, Pedersen L. Pharmacy-based Medical Record Linkage Systems. In: Strom B, Kimmel S, Hennessy S, eds. Pharmacoepidemiology. 5 ed: John Wiley & Sons, Ltd.; 2012: 270-86.
5. van Herk-Sukel MP, van de Poll-Franse LV, Lemmens VE, et al. New opportunities for drug outcomes research in cancer patients: the linkage of the Eindhoven Cancer Registry and the PHARMO Record Linkage System. *Eur J Cancer* 2010; **46**(2): 395-404.
6. Dutch Hospital Data Foundation. www.dhd.nl.
7. Foundation P. The nationwide network and registry of histo- and cytopathology in the Netherlands. www.palga.nl (accessed 17 April 2018 2018).
8. Netherlands Comprehensive Cancer Organisation. Netherlands Cancer Registry. www.iknl.nl (accessed 17 April 2018 2018).
9. Houweling LM, Bezemer ID, Penning-van Beest FJ, Meijer WM, van Lingen RA, Herings RM. First year of life medication use and hospital admission rates: premature compared with term infants. *The Journal of pediatrics* 2013; **163**(1): 61-6.e1.
10. Houben E, Smits E, Pimenta JM, Black LK, Bezemer ID, Penning-van Beest FJ. Increased risk of morbidities and health-care utilisation in children born following preterm labour compared with full-term labour: A population-based study. *Journal of paediatrics and child health* 2018.
11. Houben E, Te Winkel B, Steegers EAP, Herings RMC. Dutch trends in the use of potentially harmful medication during pregnancy. *Br J Clin Pharmacol* 2020.
12. Blehar MC, Spong C, Grady C, Goldkind SF, Sahin L, Clayton JA. Enrolling pregnant women: issues in clinical research. *Women's health issues : official publication of the Jacobs Institute of Women's Health* 2013; **23**(1): e39-45.
13. Sheffield JS, Siegel D, Mirochnick M, et al. Designing drug trials: considerations for pregnant women. *Clin Infect Dis* 2014; **59 Suppl 7**(Suppl 7): S437-S44.
14. Perined. Perinatale Zorg in Nederland 2016. Utrecht: Perined; 2018.

For peer review only

For peer review only - http://bmjopen.bmj.com/site/about/guidelines.xhtml

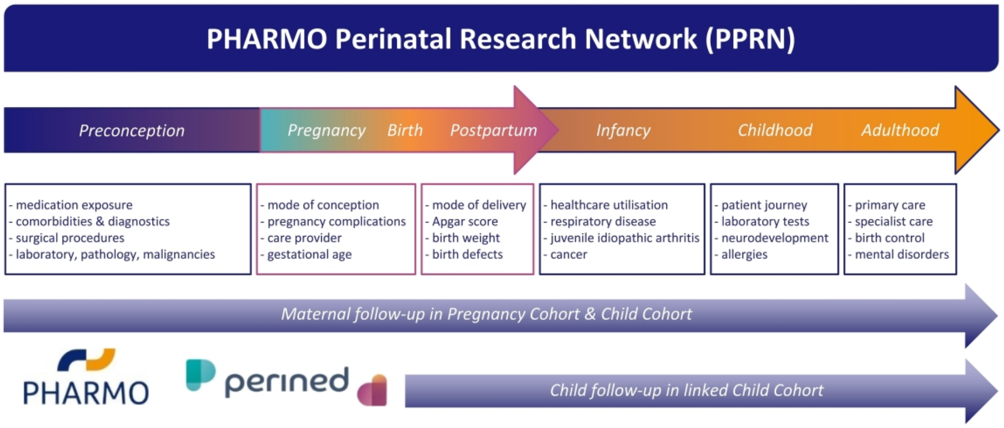

Figure 1. Schematic overview of data captured in the PHARMO-Perined linked PPRN

450x191mm (300 x 300 DPI)

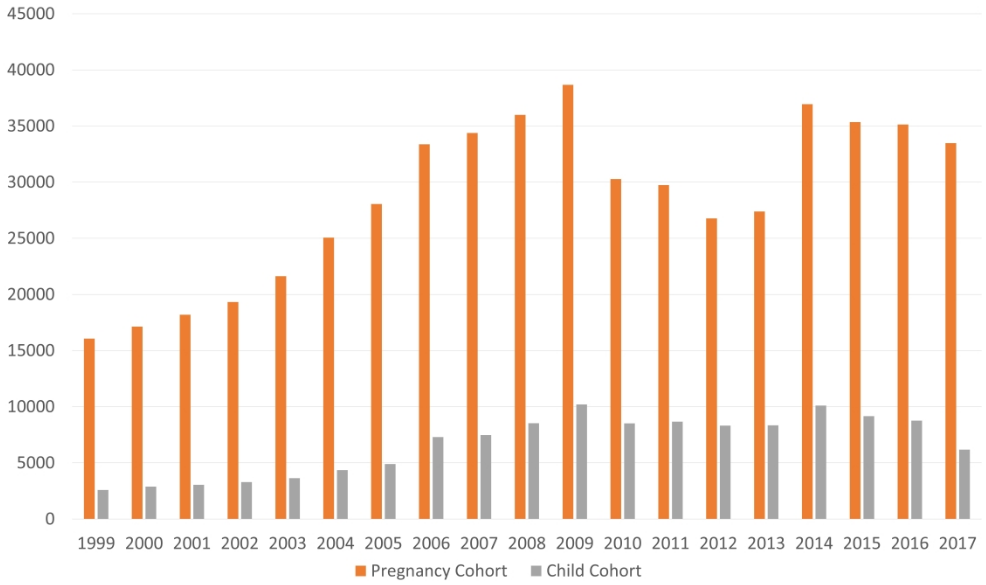

Figure 2. Number of pregnancies included in the Pregnancy Cohort and Child Cohort by calendar year

599x360mm (300 x 300 DPI)

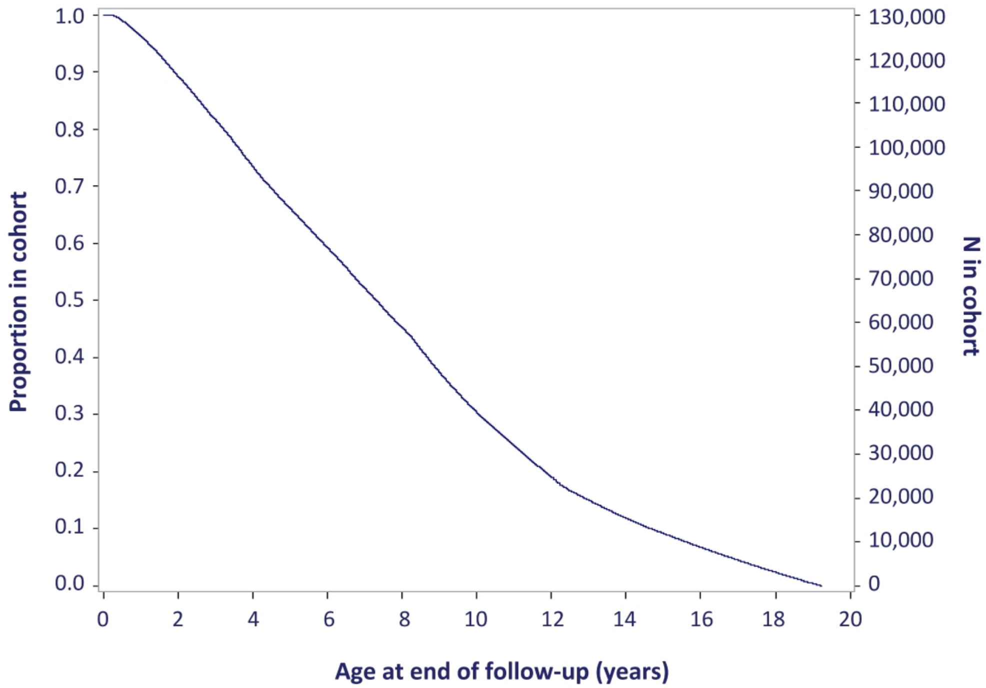

Figure 3. Proportion and number of children included in the PPRN Child cohort, with age in years at end of follow-up (current censoring: 31 December 2018)

556x396mm (300 x 300 DPI)

Appendix 1. Citation list for work published on the PPRN (excluding conference abstracts)

- Houweling LM, et al.: First year of life medication use and hospital admission rates: premature compared with term infants. *J Pediatr* 163(1): 61-6.e1, 2013.
- Zomerdijk IM, et al.: Isotretinoin exposure during pregnancy: a population-based study in The Netherlands. *BMJ Open* 4(11): e005602, 2014.
- Zomerdijk IM, Houweling L. Uitgifte van mogelijk teratogene geneesmiddelen voorafgaand aan en tijdens de zwangerschap. Perinatale Registratie Nederland. Grote Lijnen 1999 - 2012. 2014:p74-75.
- Kool-Houweling LM, et al.: Infants born before 32 weeks of gestation or with respiratory disease are most likely to receive palivizumab in the Netherlands. *Acta Paediatr* 104(9): 927-32, 2015
- Zomerdijk IM, et al.: Dispensing of potentially teratogenic drugs before conception and during pregnancy: a population-based study. *Bjog* 122(8): 1119-29, 2015.
- Houben E, et al.: Increased risk of morbidities and health-care utilisation in children born following preterm labour compared with full-term labour: A population-based study. *J Paediatr Child Health*, 2018.
- Molenaar NM, et al.: Dispensing patterns of selective serotonin reuptake inhibitors before, during and after pregnancy: a 16-year population-based cohort study from the Netherlands. *Arch Womens Ment Health*, 2019

For peer review only
